# Editorial: Food Loss and Waste: Not All Food Waste Is Created Equal

**DOI:** 10.3389/fnut.2021.615550

**Published:** 2021-06-02

**Authors:** Namy Espinoza Orias, Christian John Reynolds, Alexi Sara Ernstoff, Ian Vázquez-Rowe, Karen Cooper, Rubén Aldaco

**Affiliations:** ^1^Societé des Produits Nestlé S.A., Lausanne, Switzerland; ^2^Centre for Food Policy, School of Health Sciences, City, University of London, London, United Kingdom; ^3^Quantis SàRL, Lausanne, Switzerland; ^4^Peruvian LCA Network, Department of Engineering, Pontificia Universidad Católica del Perú, Lima, Peru; ^5^Societé des Produits Nestlé S.A., Vevey, Switzerland; ^6^Department of Chemical and Biomolecular Engineering, Cantabria University, Santander, Spain

**Keywords:** food loss and waste, measurement, methods, value, multiple valuation approach, food system

Action on reducing food loss and waste (FLW) is imperative to mitigate the impacts of climate change worldwide and to achieve the Sustainable Development Goals on food security, hunger eradication, and sustainable production and consumption. Next year (2022) will be the UN summit of food systems (“the COP for food”), as articles in this special issue illustrate, FLW will stay as one of the major levers to pull for food system transformation and sustainable consumption.

The objective of this Research Topic is to go beyond a linear approach to FLW and to look at it from a systemic perspective, measuring it not just using the standardized metric of mass but multiple valuation frameworks. Considering nutritional value, environmental impact, social impact, costs (explicit and hidden), or potential for nutrient recycling, and the various points of view of the stakeholders in the food system. The premise is that better understanding of FLW and potential interventions and solutions will come from multiple and complementary ways of analyzing and valuing it. In this editorial we summarize the papers in the Research Topic and end with a call to action.

Toti et al. present the novel perspective of “metabolic food waste” i.e., overeating. One hundred and forty gigatons of food was found to be metabolically wasted globally, mostly driven by North America and Europe. Dairy, milk, and eggs were key over consumed products that also have relatively high greenhouse gas intensity (kg CO_2_-eq per kilogram) suggesting future work can focus on balancing consumption of these products.

Horton et al. reframe FLW as the result of process inefficiencies across the entire agri-food value chain, giving rise to overall food chain inefficiency. Nine conversion efficiency factors are presented, some of them already regularly calculated -such as harvesting or processing efficiency- and others only now gathering salience, such as consumption efficiency or dietary efficiency (the latter is addressed also in this Research Topic by Toti et al.). The approach is illustrated by evaluating the bread supply chain in the UK. Ultimately, providing sustainable food security to humankind depends absolutely upon increasing the efficiency of the agri-food system, whilst at the same time ensuring it is resilient, resourceful, and environmentally sustainable. This can be achieved if we first establish a uniform approach to analyse each step in the system, as begun here.

van Dooren et al. provided valuable insight into a practical solution to tackling FLW at a household level 1.6 million “*Eetmaatje* Cups” were made available to Dutch households to measure portions for pasta and rice, two foods often overestimated in volumes before cooking, which not only can be wasted but could lead to overconsumption. Users of the cup self-reported less FLW of these two foods, with measured losses trending downwards. These types of practical solutions should be highly welcomed and, when used in conjunction with education and awareness, will need to be part of the solution space for the complex issue of household FLW.

March et al. provide a straightforward evaluation of FLW in fresh milk, paired with identification and measurement of causes at farm level and carbon footprinting. This paper highlights the possible application of Horton et al., where inefficiencies at farm level are proposed. This paper also contributes to the literature by contributing and corroborating primary data for dairy FLW. We encourage this work to be carried out for all value chains, all archetypes, and all regions.

von Massow et al. consider the nutritional, economic, and environmental impacts of avoidable FLW in Canadian households. Nearly 70% of FLW in the surveyed households was classified as avoidable and was largely composed of fruits and vegetables. There was a large variation of wastes between households suggesting future work can focus on interventions for a subset of consumers.

This article by Isah and Ozbay provides a review of FLW valorisation, with the focus on biofuels and chemicals products. It adds to the growing body of literature that highlights the potential of these technologies to help recapture value, minimize impacts, and create new products from FLW.

Goossens et al. highlight the importance of bringing together FLW prevention and treatment actions with the final purpose of indexing the performance of these measures. For this, the effectiveness of reducing FLW throughout supply chains should be accompanied by monitoring the actions established with a set of economic, social, and environmental indicators, which are currently treated in an uneven manner in the literature. This way they argue that effective FLW minimization can be coupled with metrics that guarantee an efficient treatment of monetary, environmental, or nutritional aspects, avoiding unwanted trade-offs between indicators. Therefore, the authors advocate for the implementation of harmonized evaluation criteria to consistently determine the appropriateness of FLW prevention and mitigation schemes.

Winans et al. analyze the food loss that occurs at a farm in California from an LCA perspective. They compare the conventional production of 4 different specialty crops considering optimal harvest with the integration of food losses and the treatment (e.g., energy from biomass) or recovery of these losses. Environmental impacts showed to be highly dependent on the food loss ratio reported for each crop as well as on the circularity measures adopted to recover these losses.

The papers in this special issue present FLW not just using the standardized metric of mass but multiple valuation frameworks. These now sit alongside multiple other reports (1–3) and peer reviewed publications providing powerful evidence that globally, nationally, and locally, we need FLW reduction and minimization. However, it is telling that only one of our papers evaluates an intervention (the *Eetmaatje* measuring cup). To reduce FLW (and to achieve SDG12.3), we now need to go beyond measurement to test interventions, and provide peer reviewed evidence that reductions are possible, with solutions and evaluations provided. Circular and bio- economy are emerging as key concepts for sustainable development. Assessing the consequences of targets to reduce food waste and at the same time improve destination management (e.g., anaerobic digestion and composting) within a circular economy could be an interesting area of future work that was not covered in the submissions to this special issue.

## Author Contributions

All authors contributed with the review of manuscripts and preparation of this Editorial.

## Conflict of Interest

NE and KC were employed by company Societé des Produits Nestlé S.A. AE was employed by Quantis SàRL. The remaining authors declare that the research was conducted in the absence of any commercial or financial relationships that could be construed as a potential conflict of interest.
